# Association of Tea Consumption with Risk of Alzheimer’s Disease and Anti-Beta-Amyloid Effects of Tea

**DOI:** 10.3390/nu10050655

**Published:** 2018-05-22

**Authors:** Curt Anthony Polito, Zhuo-Yu Cai, Yun-Long Shi, Xu-Min Li, Rui Yang, Meng Shi, Qing-Sheng Li, Shi-Cheng Ma, Li-Ping Xiang, Kai-Rong Wang, Jian-Hui Ye, Jian-Liang Lu, Xin-Qiang Zheng, Yue-Rong Liang

**Affiliations:** 1 Tea Research Institute, Zhejiang University, Hangzhou 310058, China; curtpolito@outlook.com (C.A.P.); 21716160@zju.edu.cn (Z.-Y.C.); 11516051@zju.edu.cn (Y.-L.S.); 21616096@zju.edu.cn (X.-M.L.); 21616106@zju.edu.cn (R.Y.); 11616052@zju.edu.cn (M.S.); qsli@zju.edu.cn (Q.-S.L.); jianhuiye@zju.edu.cn (J.-H.Y.); jllu@zju.edu.cn (J.-L.L.); xqzheng@zju.edu.cn (X.-Q.Z.); 2 Liupao Tea Academy, Wuzhou 543003, Guangxi, China; zjumasc@aliyun.com; 3 National Tea and Tea product Quality Supervision and Inspection Center (Guizhou), Zunyi 563100, China; gzzyzj_2009@vip.sina.com; 4 Ningbo Extension Station of Forestry & Specialty Technology, Ningbo 315012, China; wkrtea321hjytea@163.com

**Keywords:** *Camellia sinensis*, epigallocatechin gallate (EGCG), theanine, caffeine, Alzheimer’s disease, Parkinson’s disease

## Abstract

Neurodegenerative disease Alzheimer’s disease (AD) is attracting growing concern because of an increasing patient population among the elderly. Tea consumption is considered a natural complementary therapy for neurodegenerative diseases. In this paper, epidemiological studies on the association between tea consumption and the reduced risk of AD are reviewed and the anti-amyloid effects of related bioactivities in tea are summarized. Future challenges regarding the role of tea in preventing AD are also discussed.

## 1. Introduction

Alzheimer’s disease (AD) is progressive neurodegenerative disorder pathologically characterized by deposition of β-amyloid (Aβ) peptides as senile plaques in the brain and its prevalence is strongly correlated with aging [1]. AD is the second leading health concern among adults following cancer [2], being the sixth leading cause of death, and also the only disease among the top 10 that cannot be prevented, cured, or treated [3]. AD is characterized by a progressive cognitive decline, leading to dementia [4]. The increase in life expectancy due to modern society and the associated healthcare has been accompanied by an increase in the number of people with AD. It is estimated that 50% of people with aged 85 or older suffered from AD [5]. In the United States, someone develops AD every 67 seconds [3]. In China, 7.4 million elderly persons are estimated to have dementia, and this number is expected to grow to 18 million by 2030 if effective preventions are not identified and implemented [6]. Although many AD-related treatment hypotheses have been proposed, the exact causes and pathogenesis of AD are still unclear. Furthermore, along with other neurodegenerative dementias diseases, AD lacks any effective cure. For this reason, the prevention of AD and non-pharmacological treatments are important research [7].

Dietary interventions might play a role in the prevention of AD. Beverages containing plant polyphenolshave been recommended as a natural complementary therapy for alleviating the symptoms of AD [8]. Specifically, one study reported that language and verbal memory were positively associated with the intake of green tea catechins and black tea theaflavins [9]. Data from several cross-sectional studies consistently showed that tea drinking is associated with better performance on cognitive tests. Tea consumption is considered to be one simple lifestyle adjustment that may either prevent or treat the cognitive declines associated with neurodegenerative AD [10,11]. 

Many review articles focused on the subject of tea polyphenols and potential neuroprotective properties, in which the potential benefits of tea catechins for reducing the risk of AD by targeting the effects of oxidation, iron chelating, microglia activation, andmodulating intracellular neuronal signal transduction pathways [12,13,14]. The originality of the present review includes two aspects: (1) the neurodegenerative process in AD is characterized by the presence of cerebral extracellular deposition of Aβ and the published reviews rarely focused on the anti-Aβ effects of tea. The present review summarizes the advances in the anti-Aβ effects of tea with regards to its association with AD. (2) The latest review of the association of tea with AD updated the literature published until December 2016 [14]. Since then, more than 10 research papers have been published on this topic that involved epidemical surveys and mechanism studies. The most significant research advances regarding tea’s potential role in the prevention and treatment of AD and other related neurodegenerative symptoms were included in the present review by searching the Web of Science database using keywords “tea” and “Alzheimer’s disease” and the cited references were updated until February 2018. 

## 2. Epidemiological Evidence

Considerable epidemiological evidence has associated tea consumption with a decreased risk of AD and other neurodegenerative diseases. The procedure for preparing a cup of tea was used to assess the action-based memory of people with AD dementia [15]. In Japan, a community-based comprehensive geriatric assessment involving 1003 Japanese residents aged 70 or older showed that a higher consumption of green tea was associated with a lower prevalence of cognitive impairment (CoI). At the cutoff cognitive function score of below 26 as evaluated by the Mini-Mental State Examination (MMSE), the odds ratios (OR) were 0.62 (95% confidence interval (95% CI): 0.33, 1.19) for four to six cups per week to one cup per day and 0.46 (95% CI: 0.30, 0.72) for two or more cups per day (*p* = 0.0006), compared to the OR = 1.00 for reference (≤3 cups/week) [16]. A cohort study involving 13,988 Japanese people aged 65 or older showed that green tea consumption was significantly associated with a lower risk of incident functional disability, among which the three-year incidence of functional disability was 9.4% (1316 cases). The multiple-adjusted hazard ratio (HR) of the incidentfunctional disability was 0.90 (95% CI: 0.77, 1.06) among respondents who consumed one to two cups of green tea per day, 0.75 (95% CI: 0.64, 0.88) for those who consumed three to four, and 0.67 (95% CI: 0.57, 0.79) for those who consumed five or more cups per day, in comparison with those who consumed one or fewer cups/day (*p* = 0.001) [17]. A follow-up 4.9 ± 0.9 years’ population-based prospective study with 490 Japanese residents aged 60 or older from Nakajima showed that the multiple-adjusted ORs for the incidence of overall cognitive decline (MCI) was 0.32 (95% CI: 0.16, 0.64) among individuals who consumed green tea every day and 0.47 (95% CI: 0.25, 0.86) among those who consumed green tea one to six days per week, compared with individuals who did not consume green tea at all. The multiple adjusted OR for the incidence of dementia was 0.26 (95% CI: 0.06, 1.06) among individuals who consumed green tea every day, compared with those who did not consume any green tea. No association was found between the consumption of coffee or black tea and the incidence of dementia or MCI [18]. A cross-sectional study including 1143 Japanese residents showed that low green tea consumption was independently associated with a higher prevalence of CoI (*p* = 0.032), with an OR for drinking tea daily of 0.65 (95% CI: 0.47, 0.89) [19]. However, a double-blind randomized controlled study involving 33 nursing home residents revealed that consumption of 2 grams per day of green tea powder for 12 months was not significantly associated with cognitive disfunction, compared with that of the placebo group (OR: −0.61 (95% CI: −2.97, 1.74, *p* = 0.59)) [20].

In Singapore, a cross-sectional study involving 2501 participants aged 55 or older showed that regular tea consumption was associated with a lower risk of CoI. Compared with the ORs for rare or no tea consumption, the ORs for low (<1 cup/day), medium (1–5 cups/day), and high levels (≥6 cups/day) of tea consumption were 0.56 (95% CI: 0.40, 0.78), 0.45 (95% CI: 0.27, 0.72), and 0.37 (95% CI: 0.14, 0.98), respectively (*p* < 0.001) [21]. Another cross-sectional study involving 716 adults aged 55 or older showed that the protective effect of tea consumption on cognitive function was not limited to a particular type of tea. Total tea consumption was independently associated with better performance on global cognition (regression coefficient (B) = 0.055, standard error (SE) = 0.026, *p* = 0.03), memory (B = 0.031, SE = 0.012, *p* = 0.01), executive function (B = 0.032, SE = 0.012, *p* = 0.009), and information processing speed (B = 0.04, SE = 0.014, *p* = 0.001) based on the MMSE total score. Both black and oolong tea and green tea consumption were associated with better cognitive performance. However, no association was found between coffee consumption and cognitive function [22]. A longitudinal aging study involving 1615 adults aged 55 to 93 examining the association between the amount of tea drinking and incident depressive symptoms from follow-up over an average period of 18 months showed that the proportion of participants with depression at the follow-up was 6.6% for participants with no tea consumption, 5.3% for low tea consumption participants (<1 cup/day), 3.2% for medium tea consumption participants (1–5 cups/day), and 1.8% for high tea consumption participants (≥6 cups/day). The ORs were 0.79 (95% CI: 0.42, 1.48) for low tea consumption participants, 0.47 (95% CI: 0.25, 0.88) for medium tea consumption participants, and 0.27 (95% CI: 0.11, 0.63) for high tea consumption participants (*p* = 0.01) [23]. A cohort study involving 614 adults aged 60 or older who were free of dementia and CoI showed that long-term tea consumption for at least 15 years was associated with reduced depressive and anxiety symptoms among community-living elderly persons [24].

In China, a cohort study revealed that among 681 unrelated Chinese aged 90 or older (67.25% women), men with CoI had significantly lower prevalence of tea drinking (*p* = 0.041 and 0.044, for former and current tea drinking, respectively); whereas in women, CoI was not associated with tea drinking [25]. A national population-based prospective nested case-control study involving 5691 elderly residents aged 65 or older showed an inverse association between tea drinking and cognitive decline (OR: 0.82; 95% CI: 0.69, 1.00, *p* = 0.0468) [26]. A town level population-based survey involving 4579 persons aged 60 or older from Weitang in Suzhou City showed that tea consumption was inversely associated with the prevalence of CoI (OR: 0.74, 95% CI: 0.57, 0.98, *p* = 0.032). The protective correlation of tea was more obvious in persons who never smoked (OR: 0.63) but vanished in current or former smokers (OR: 1.10) [27]. A rural population-based study involving 1368 rural community-dwelling individuals aged 60 or older (59.3% women) showed that daily tea consumption was associated with a lower likelihood of depressive symptoms in older people in rural communities. The association appeared to be independent of cerebrovascular disease and atherosclerosis. The ORs of having high depressive symptoms were 0.86 (95% CI: 0.56, 1.32) for weekly and 0.59 (95% CI: 0.43, 0.81) for daily tea consumption (*p* = 0.001) [28]. Another study involving 9375 persons aged 60–65 and 2015 persons aged 65 or older showed that tea consumption was inversely correlated with prevalence of CoI [29] and AD [30]. Data from the Chinese Longitudinal Healthy Longevity Surveys showed that drinking tea had a positive impact on cognitive function. A survey involving 32,606 individuals (13,429 men and 19,177 women) aged 65 or older showed that frequent tea consumption was significantly associated with reduced OR of CoI [31]. Another survey involving 7139 participants aged 80–115 years showed that regular tea drinking was associated with better cognitive function among the oldest of the living Chinese persons. In a linear mixed effects model that adjusted for age, gender, years of schooling, physical exercise, and activities, the regression coefficient was 0.72 (*p* < 0.0001) for daily drinking and 0.41 (*p* = 0.01) for occasional drinking. Tea drinkers had higher verbal fluency scores throughout the follow-up period but concurrently had a steeper slope of cognitive decline compared with non-drinkers [32]. A prevalence survey involving 1000 residents aged 60 or older in which the samples were collected by the multi-stage random cluster sampling method in Huangshi City, China showed that drinking tea reduced the incidence of MCI (*p* < 0.05) [33]. However, a cross-sectional study including 870 residents aged 90 or older showed no significant correlation between tea consumption and the prevalence of MCI among this group [34].

In Norway, a cross-sectional study involving 2031 participants aged 70–74 (55% women) showed that participants who consumed chocolate, wine, or tea had significantly lower prevalence of poor cognitive performance than those who did not. Participants who consumed all three tested items had the best cognitive testing scores and the lowest risks for poor cognitive testing performance. The associations between intake of these foodstuffs and cognition were dose dependent, with an approximately linear relationship for tea consumption [35].

A large-scale population study involving participants from 23 developed countries given different genetic backgrounds found a significant inverse correlation between dietary consumption of flavonoids (also a group of polyphenols found in green tea) and disability-adjusted life year rates of AD and other related dementias [36]. A meta-analysis involving 52,503 participants from Asia, Europe, Australia, and North America showed that daily tea consumption was associated with a decreased risk of CoI, MCI, and cognitive decline in elderly persons. Tea consumption significantly reduced the risk of cognitive disorders (OR = 0.65, 95% CI: 0.58, 0.73). Tea consumption was inversely associated with the risk of CoI, MCI, cognitive decline, and other ungrouped cognitive disorders. However, another investigation also showed that the association between tea consumption and AD remained elusive [37] (Table 1).

## 3. Anti-Aβ Effects of Tea

The amyloid cascade hypothesis states that naturally occurring Aβ monomers aggregate via a nucleation-dependent pathway to form insoluble fibrils that are deposited as plaques in the brain. The self-assembly of Aβ into neurotoxic oligomers followed by fibrillar aggregates is a defining characteristic of AD. AD is characterized by misfolding, aggregation, and accumulation of amyloid fibrils in an insoluble form in the brain. Green tea polyphenols (GTPs) including (−)-epigallocatechin gallate (EGCG), (+)-catechin (C) and (−)-epicatechin (EC), myricetin, quercetin, and kaempferol can protect cells from Aβ-mediated neurotoxicity by dose-dependently inhibiting the formation of Aβ fibrils (fAβ) from fresh Aβ(1–40) and Aβ(1–42) through the destabilization of preformed fAβ. The effective concentrations (EC_50_) of myricetin and quercetin for the formation, extension, and destabilization of fAβ are 0.1–1.0 μM. Although the mechanisms by which these polyphenols inhibit fAβ formation from Aβ and destabilize pre-formed fAβ in vitro are still unclear, polyphenols are considered to be valuable for the prevention and therapeutic treatment of AD [38].

GTPs are believed to combat neurodegenerative diseases by inhibiting amyloid fibril formation andprotectingneurons from toxicity induced by Aβ. Okadaicacid (OA) is a toxin that inducesneurotoxicity. GTPs considerablyreducedprimary hippocampal neurondamage induced by OA. In mice pretreated with OA, ethologic tests indicated that the staying time and swimming distance in the target quadrant significantly decreased, whereas mice pretreated with GTPs stayed longer in the target quadrant [39]. In “Swedish” mutant Aβ precursor protein (APP) over expressing mice (APPsw, Tg), intraperitoneal (i.p.) injection (20 mg/kg) of green tea EGCG decreased Aβ levels and plaques via promotion of the non-amyloidogenic α-secretase proteolytic pathway. Oral administration of 50 mg/kg EGCG in drinking water reduced Aβ deposition in the tested mice. A six-month EGCG treatment revealed that plaque burdens decreased in the cingulate cortex, hippocampus, and entorhinal cortex by 54, 43, and 51%, respectively. Congo red plaque burden were decreased in the cingulate cortex, hippocampus, and entorhinal cortex by 53, 53, and 58%, respectively, and were accompanied by a reduction in both Aβ(1–40) and Aβ(1–42). Radial Ann water maze (RAWM) testing for working memory indicated that EGCG provided a cognitive benefit to Tg mice with both i.p. and oral administration; however, i.p. treated benefited more [40]. The anti-Aβ mechanism of tea is summarized below.

### 3.1. Inhibiting APP Cleavage by Regulating Activity of Related Enzymes

EGCG reduced Aβ generation in both murine neuron-like cells (N2a) transfected with Swedish mutant APP mice and primary neurons derived from Swedish mutant APP-overexpressing mice (Tg APPsw line 2576). EGCG markedly promoted cleavage of the α-C-terminal fragment of APP and elevated the N-terminal APP cleavage product, soluble APP-α. These cleavage events are associated with elevated α-secretase activity and enhanced hydrolysis of tumor necrosis factor α-converting enzyme, a primary product of α-secretase. In vivo tests on Tg APPsw transgenic mice showed that EGCG administration decreased Aβ levels and plaques by promotingthe nonamyloidogenic α-secretase proteolytic pathway [41,42].

The β-site APP cleaving enzyme 1 (BACE1) is a rate-limiting enzyme in APP processing and Aβ generation. The nuclear receptor peroxisome proliferator-activated receptor γ (PPARγ) is a potential target for AD treatment because of its potent inhibitory effects on Aβ production by negatively regulating BACE1. EGCG reduced Aβ generation in N2a/APP695 cells similar to the PPARγ agonist pioglitazone by inhibiting the transcription and translation of BACE1. This effect was reduced by the PPARγ inhibitor GW9662. EGCG significantly reinforced the activity of PPARγ by promoting its mRNA and protein expressions. The therapeutic efficacy of EGCG in testing for AD is thought to be derived from the up-regulation of PPARγ mRNA and protein expressions [43]. EGCG modulated APP processing, which resulted in enhanced cleavage of the α-COOH-terminal fragment (α-CTF) of APP and the corresponding elevation of the NH2-terminal APP product [i.e., soluble APP-α (sAPP-α)]. These beneficial effects were associated with increased α-secretase cleavage activity. Furthermore, EGCG treatment markedly elevated active ADAM10 protein (a-disintegrin and metalloprotease) in N2a cells by increasing α-CTF cleavage and elevating sAPP-α.

ADAM10 is an important pharmacotherapeutic target for the treatment of cerebral amyloidosis in AD. ADAM10 activation is critical for EGCG promotion of non-amyloidogenic (α-secretase cleavage) APP processing [44]. Estrogen depletion following menopause has been correlated with an increased risk of developing AD. EGCG increased non-amyloidogenic processing of APP through ADAM10, which was mediated by the maturation of ADAM10 via an estrogen receptor-α/phosphatidylinositol 3-kinase/aserine/threonine-specific protein kinase (ERα/PI3K/Akt) signaling-dependent mechanism, independent of furin-mediated ADAM10 activation. Central selective ER modulation could be a therapeutic target for AD, and EGCG could be used as a well-tolerated alternative to estrogen therapy in the prophylaxis and treatment of this disease [45]. Oral administration of EC, another type of tea catechin, showed the same effect on Aβ pathology by inhibiting BACE1 [46].

Prolyl endopeptidase (PEP) is a serine protease known to cleave peptide substrates on the C-terminal side of proline residues. PEP also plays an important role in the degradation of proline-containing neuropeptides such as oxytocin, vasopressin, substance P, neurotensin, and angiotensin, which have been suggested as participants in the learning and memory processes [47]. The PEP activity in persons with AD was significantly higher than that in those without AD [48]. PEP could be involved in the processing of the C-terminal portion of the APP in AD [49]. Specific PEP inhibitors could prevent memory loss and increase attention span in patients suffering from senile dementia. EGCG, (−)-epicatechin gallate (ECG), and (+)-gallocatechin gallate (GCG) extracted from tea leaves were PEP inhibitors, with IC_50_ values of 1.42 × 10^–4^ mM, 1.02 × 10^–2^ mM, and 1.09 × 10^–4^ mM, respectively. They were non-competitive with a substrate in Dixon plots and did not show any significant effects on any other serine proteases like elastase, trypsin, and chymotrypsin, suggesting that they were relatively specific inhibitors against PEP and may be useful for preventing AD [50].

The drug therapies for AD are based on the cholinergic hypothesis that AD begins as a deficiency in the production of the neurotransmitter acetylcholine. Cholinesterase inhibition might impact the processing of amyloid in AD [51] and cholinesterase inhibitors have been suggested as the standard drugs for the treatment of AD. The inhibitors of acetylcholinesterase (AChE) and butyrylcholinesterase (BChE) show potential in the treatment process of AD. A molecular docking study revealed that EGCG inhibited AChE and BChE, resulting in enhance cholinergic neurotransmission [52].

Caffeine, a major component in tea, induced an increase in specific cellular neutral endopeptidase (NEP) activity in neuroblastoma cell line SK-N-SH and its activity was stronger than theophylline, theobromine, or theanine. The combination of EC, EGC, and EGCG with caffeine, theobromine, or theophylline induced cellular neutral endopeptidase activity. The enhancement of cellular NEP activity by green tea extract and its natural products might be correlated with an elevated levelofintracellular cyclic adenosine monophosphate [53].

Lipopolysaccharide (LPS) impairsmemory through the accumulation of Aβ via the increase of β- and γ-secretase. Oral treatment with EGCG (1.5 and 3 mg/kg for three weeks) into drinking water ameliorated LPS (1 μg/mouse, i.c.v.)-induced memory deficiency in a dose dependent manner. EGCG also dose-dependently inhibited LPS-induced elevation of Aβ levels by reducing LPS-induced β- and γ-secretase activities and expression of its metabolic products such as C99 and Aβ. EGCG prevented LPS-induced neuronal cell death as well as the expression of inflammatory proteins through inducible nitric oxide synthetase and cyclooxygenase. EGCG prevented LPS-mediated apoptotic cell death through suppression of Aβ elevation by inhibiting β- and γ-secretase. As a result, EGCG might be a useful agent against the neuroinflammation-associated development or progression of AD [54].

The kinetics of inhibition tests using Dixon, Cornish-Bowden, and Lineweaver-Burk plots showed that green, oolong, and black tea extracts, EGCG, theaflavin-3,3′-digallate (TFDG), and tannic acid were competitive inhibitors of PPA, whereas ECG, theaflavin-3′-gallate (TFG), and theaflavin (TF) were mixed-type inhibitors with both competitive and uncompetitive inhibitory characteristics. Only catechins with a galloyl substituent at the three-position showed a measurable inhibition. The competitive inhibition constants (Kic) were lower for theaflavins (TFs) than catechins, with the lowest value recorded for TFDG, suggesting that TFs and catechins bound more tightly with free PPA than with the PPA-starch complex. A 3 and/or 3′-galloyl moiety in catechin and TF structures was consistently found to increase the inhibition effect on PPA by enhancing association with the enzyme activation site. Various catechins showed different inhibitory effects on PPA, with IC_50_ being 2.514 mg/mL for EGCG, 1.729 mg/mL for ECG, 0.412 mg/mL for TF, 0.244 mg/mL for TFG, and 0.130 mg/mL for TFDG [55].

### 3.2. Preventing Protein Misfolding and Aβ-Induced Membrane Damages

Misfolded Aβ peptides self-assemble into higher-order oligomers that compromise membrane integrity, leading to synaptic degeneration and neuronal cell death. The misfolding of the Aβ peptide is one of the pathological hallmarks of AD. Aβ(1–42) peptides aggregated into a range of oligomers that efficiently permeabilized small unilamellar liposomes that were used to assess the ability of tea extracts to antagonize liposome permeabilization by the Aβ(1–42) oligomers. The dihydroxyphenyl ring structure of tea catechins, alone or as part of a flavanol scaffold, is particularly effective in protecting against membrane damage induced by the Aβ(1–42) oligomers [56]. Given the critical role of membrane perforation in the neurodegenerative cascade, these could guide the design and development of novel therapeutic drugs for the treatment of AD. EGCG plays special role in protein-misfolding diseases because of its potent anti-amyloid activity against Aβ, α-synuclein and huntingtin. EGCG redirected the aggregation of these polypeptides to a disordered off-folding pathway that results in the formation of non-toxic amorphous aggregates. EGCG also inhibits in vitro fibril formation via reduced and carboxymethylated kappa-casein (RCMkappa-CN), by preventing RCMkappa-CN fibril formation by stabilizing RCMkappa-CN in its native-like state. EGCG was proposed to be directed to the amyloidogenic sheet-turn-sheet motif of monomeric RCMkappa-CN with high affinity by strong non-specific hydrophobic associations, with non-covalent pi-pi stacking interactions between the polyphenolic and aromatic residues on the amyloidogenic sequence [57].

The chelating ability of EGCG also plays a role in reducing fibril formation. Observations using square wave voltammetry and transmission electron microscopy showed that the interaction of Cu(II) ions with the Tyr-10 residue of Aβ was affected by the surrounding His residues. With only Cu(II) present, the Aβ(1–40) aggregates showed a dense structure due to possible interactions within the metal binding region of Aβ(1–40) peptides. However, unstructured aggregates were observed when both EGCG and Cu(II) ions were incubated with Aβ(1–40), demonstrating that the chelating ability of EGCG impeded the formation of the Cu(II)-His complex, resulting in reduced fibril formation [58]. Both unoxidized and oxidized EGCG are active in inhibiting fibril formation, but the in vitro EGCG amyloid remodeling activity was dependent on auto-oxidation of the EGCG. Tests showed that the oxidized and unoxidized EGCG bound to amyloid fibrils, preventing the binding of thioflavin T. The hydrophobic binding sites were in A1–40, IAPP8–24, or Sup35NMAc7–16 Y→F amyloid fibrils. The oxidized EGCG molecules reacted with free amines within the amyloid fibril through the formation of Schiff bases, cross-linking the fibrils, which may prevent dissociation and toxicity [59].

### 3.3. Mitigating Aβ-Induced Oxidative Stress

Aβ peptides play a bilateral role in neuronal cell oxidative stress. Reactive oxygen species (ROS) induce formation of Aβ, which stimulates oxidative stress and neuronal toxicity. This process is typically attenuated by antioxidants and free radical scavengers. Tea catechins are a group of natural antioxidants that have protective effects against Aβ-induced neuronal apoptosis by scavenging ROS. One study recorded marked hippocampal neuronal injuries and increases in malondialdehyde (MDA) levels and caspase activity after the hippocampal neuronal cells were exposed to Aβ for 48 h. However, co-treatment of cells with EGCG to Aβ exposure increased the cell survival rate and decreased the levels of MDA and caspase activity. Proapoptotic (p53 and Bax), Bcl-XL, and cyclooxygenase (COX) proteins have been implicated in Aβ-induced neuronal death. The protective effects of EGCG are considered to be independent of the regulation of p53, Bax, Bcl-XL, and COX proteins. This suggests that EGCG has protective effects against Aβ-induced neuronal apoptosis by scavenging ROS, which is beneficial for the prevention and slowing of AD [60]. Aβ and pro-oxidant evoked neurotoxicity in PC12 cells, which resulted in a concentration-dependent reduction in viability of PC12 cell and human SH-SY5Y neuroblastoma cells via multiple protection mechanisms including the reduction of the pro-apoptotic proteins and Bax, the decrease in apoptosis-associated Ser139 phosphorylated H2A.X, and inhibition of the cleavage and activation of caspase-3. EGCG significantly reduced Aβ-evoked neurotoxicity [61,62].

EGCG may have preventive and/or therapeutic potential in AD patients by augmenting cellular antioxidant defense capacity and attenuating Aβ-mediated oxidative and/or nitrosative cell death. Aβ-induced damage of the neurons and glia are mediated via nitrosative and oxidative stress. BV2 cells exposed to Aβ underwent nitrosative stress, as shown by the increased expression of inducible nitric oxide synthase (iNOS) and subsequent production of nitric oxide (NO) and peroxynitrite, which were effectively suppressed by EGCG pretreatment. The mechanism considered to be at work is EGCG treatment fortifying the cellular GSH pool through elevated mRNA expression of γ-glutamylcysteine ligase, a rate limiting enzyme in glutathione biosynthesis [63]. Tea polyphenols EGCG, EC, and TF suppressed oxidative stress-induced BACE-1 mRNA upregulation in neuronal cells, resulting in the reduction of amyloidogenic cleavage of APP and Aβ production [3,64]. Green tea extracts protected neuronal dPC12 cells from H_2_O_2_-induced and Aβ-induced cytotoxicity at concentration ranges of 0.3–10 μg/mL and 0.03–0.125 μg/mL, respectively [65].

Aβ fragment individuals caused neurotoxicity through oxidative stress. Partial tea components and/or their complexes with Aβ fragments showed antioxidative activity. Injection of Aβ(25–35) (100 μM/μL) into the CA1 hippocampal region of mice caused a significant increase in lipid peroxidation and ROS, resulting in a decrease in memory skills. Hippocampal tissues from Aβ(25–35)-treated mice showed an increased immune reactivity against glial-fibrillar acidic protein. In contrast, mice pretreated with green tea EC (30 mg/kg) had a significant decrease in lipid peroxidation and ROS, as well as an improvement in memory skills. This result shows that Aβ(25–35)-caused oxidative damage in the hippocampus was blocked by the administration of EC [66]. CA-Aβ(38–42), a complex of the antioxidant caffeic acid (CA) and Aβ, exhibited potent inhibitory activity against Aβ(1–42) aggregation and scavenged Aβ(1–42)-induced intracellular oxidative stress. CA-Aβ(38–42) also significantly protected human neuroblastoma SH-SY5Y cells against Aβ(1–42)-induced cytotoxicity, with an IC_50_ of 4 μM, suggesting that CA-Aβ(38–42) has potential for AD prevention [67].

### 3.4. Suppressing Aggregation of Aβ Oligomers and Formation of Aβ Fibrils

One of the key factors in the development of AD is the conversion of Aβ from its soluble random coil form into various aggregated forms. EGCG may play an important role in APP secretion and protection against toxicity induced by Aβ. EGCG enhanced the release of the non-amyloidogenic soluble amyloid precursor protein (sAPPα) into the conditioned media of human SH-SY5Y neuroblastoma cells and rat pheochromocytoma PC12 cells. Treatment with EGCG reduced the Aβ levels by enhancing endogenous APP nonamyloidogenic proteolytic processing. EGCG also decreased nuclear translocation of c-Abl and blocked the amyloid precursor protein fragment (APP-C99)-dependent GSK3 β activation. These inhibitory effects occurred via the interruption of c-Abl/Fe65 interaction [68].

Islet amyloid polypeptide (IAPP, amylin) lacks a well-defined structure in its monomeric state, but readily assembles to form amyloid. Amyloid fibrils formed from IAPP, intermediates generated in the assembly of IAPP amyloid, or both, are toxic to β-cells. EGCG inhibited unseeded amyloid fibril formation and disaggregated IAPP amyloid, which protected cultured rat INS-1 cells against IAPP-induced toxicity [69]. EGCG effectively reduced the cytotoxicity of Aβ by remodeling seeding-competent Aβ oligomers into off-pathway seeding-incompetent Aβ assemblies.

During the initial EGCG-Aβ interactions, EGCG interfered with the aromatic hydrophobic core of Aβ and the EGCG-induced Aβ oligomers adopted a well-defined structure. The C-terminal part of the Aβ peptide (residues 22–39) adopted a β-sheet conformation, whereas the N-terminus (residues 1–20) was unstructured. The characteristic salt bridge involving residues D23 and K28 is present in the structure of these oligomeric Aβ aggregates [70]. The remodeling adhered to a Hill-Scatchard model where by the Aβ(1–40) self-association occurred cooperatively and generated Aβ(1–40) oligomers with multiple independent binding sites for EGCG with a Kd 10-fold lower than that for the Aβ(1–40) monomers. Upon binding to EGCG, the Aβ(1–40) oligomers were less exposed to solvents, and the β-regions, which were involved in direct monomer-protofibril contacted intheabsence of EGCG, underwent a direct-to-tethered contact shift. This switch toward less engaged monomer-protofibril contacts explained the seeding incompetency observed upon EGCG remodeling and suggested that EGCG interferes with secondary nucleation events known to generate toxic Aβ assemblies. The N-terminal residues experienced an opposite EGCG-induced shift from tethered to direct contacts, explaining why EGCG remodeling occurred without release of Aβ(1–40) monomers. Upon binding Aβ(1–40) oligomers, the relative positions of the B and D rings of EGCG changed with respect to that of ring A [71]. The binding stoichiometry N is linearly related to the EGCG/Aβ42 ratio. Hydrophobic interaction and hydrogen bonding are both essential in the binding process, but the extent of their contributions changes with experimental conditions. Namely, the predominant interaction gradually shifts from a hydrogen bonding to a hydrophobic interaction with the increase in the EGCG/Aβ42 ratio, resulting in a transition of the binding from enthalpy-driven to entropy-driven. The binding of EGCG to Aβ42 can be promoted by increasing temperature and salt concentration as well as changing pH away from Aβ42’s pI [72].

l-theanine, an amide found in tea, inhibited Aβ(1–42)-induced generation of ROS and activation of extracellular signal-regulated kinase and p38 mitogenic activated protein kinase, as well as the activity of nuclear factor kappa-B. l-theanine (10–50 μg/mL) concomitantly decreased Aβ(1–42)-induced neurotoxicity in SK-N-MC and SK-N-SH human neuroblastoma cells, indicating that l-theanine prevented oxidative damages of neuronal cells and Aβ-induced neurotoxicity, which may be useful in the prevention and treatment of neurodegenerative disease like AD [73].

TFs (TF, TFG, and TFDG) had suppressive effects on Aβ aggregation, but compared to catechins, they showed different inhibitory capabilities at different mechanistic steps of the Aβ aggregation pathway. Catechins only affect the later stages of aggregation, in which catechins may bind a specific structure present in aggregates. Conversely, TFs show inhibitory capabilities at every stage of aggregation, alluding to a sequence-specific recognition. The number of gallate groups was positively correlated with inhibitory capabilities [74]. Solution-state nuclear magnetic resonance (NMR) showed that EGCG nonspecifically bound to the Aβ monomers [75]. Black tea polyphenolic component TF is a potent inhibitor of Aβ and α-synuclein (αS) fibrillogenesis. The binding regions of TFDG, congo red, and EGCG bound to two regions of the Aβ peptides, amino acids 12–23 and 24–36, albeit with different specificities. However, their mechanisms of amyloid inhibition differ. Like EGCG but unlike congo red, TFs stimulate the assembly of Aβ and αS into nontoxic, spherical aggregates that are incompetent in seeding amyloid formation and remodel Aβ fibrils into nontoxic aggregates. Compared to EGCG, TFDG was less susceptible to air oxidation and had an increased efficacy under oxidizing conditions [76].

### 3.5. Regulating Signaling Pathways Involving Aβ Generation

The EGCG-induced sAPPα secretion is blocked by the inhibition of protein kinase C (PKC). Therefore, the secretion process is considered to be PKC-dependent. EGCG shows protective effects against Aβ-induced neurotoxicity and regulates secretory processing of sAPPα via the PKC pathway. Administration of EGCG (2 mg/kg) to mice for 7 or 14 days significantly decreased membrane-bound holoprotein APP levels, with a concomitant increase in sAPPα levels in the hippocampus. EGCG markedly increased PKCα and PKε in the membrane and the cytosolic fractions of mice hippocampus. Here, EGCG was not only able to protect but also rescue PC12 cells against the Aβ toxicity in a dose-dependent manner [77]. EGCG markedly strengthened activation of α7 nicotinic acetylcholine receptor (α7nAChR) as well as its downstream pathway signaling molecules PI3K and Akt, subsequently leading to suppression of Bcl-2 downregulation in Aβ-treated neurons. Administration of α7nAChR antagonist methyllycaconitine (MLA, 20 μM) to neuronal cultures significantly attenuated the neuroprotection of EGCG against Aβ-induced neurotoxicity.

The α7nAChR activity, together with PI3K/Akt transduction signaling, may contribute to the molecular mechanism underlying the neuroprotective effects of EGCG against Aβ-induced cell death [78]. The deposition of Aβ peptides is closely correlated with the balance of nerve growth factor (NGF)-related TrkA/p75(NTR) signaling. In APP/PS1 mice, EGCG treatment (2 mg/kg·day) dramatically improved the CoI, reduced the over expression of Aβ(1–40) and APP, and inhibited neuronal apoptosis. EGCG also enhanced the relative expression level of NGF by increasing the NGF/proNGF ratio in APP/PS1 mice. After EGCG treatment, TrkA signaling was activated by increasing the phosphorylation of TrkA following the increased phosphorylation of the c-Raf, ERK1/2, and cAMP response to element-binding protein (CREB). Simultaneously, p75(NTR) signaling was significantly inhibited by decreasing the p75(ICD) expression, JNK2 phosphorylation, and cleaved-caspase 3 expression, resulting in inhibition of the Aβ deposits and neuronal apoptosis in the hippocampus [79].

Neprilysin (NEP) is an important Aβ-degrading enzyme in the brain; thus, defective enzyme expression may facilitate Aβ deposition in sporadic late onset AD patients. Treatment of cultured rat astrocytes with EGCG significantly reduced the expression of NEP in a concentration- and time-dependent manner. NEP expression in cultured astrocytes was suppressed by activation of extracellular signal-regulated kinase (ERK) and PI3K. Reduced NEP expression was accompanied by an increase in NEP release into the extracellular medium. The culture medium from EGCG-treated astrocytes facilitated the degradation of exogenous Aβ, suggesting that EGCG may have a beneficial effect on persons with AD by activating ERK- and PI3K-mediated pathways in astrocytes, thereby increasing astrocyte secretion of NEP and facilitating degradationofAβ [80].

l-theanine in tea also plays a role in regulating the signaling pathway related to Aβ deposits. Oral administration of l-theanine (2 and 4 mg/kg) to mice for five weeks in the drinking water, followed by injection of Aβ(1–42) (2 μg/mouse, i.c.v.), significantly alleviated Aβ(1–42)-induced memory impairment. l-theanine decreased Aβ(1–42) levels and the accompanying Aβ(1–42)-induced neuronal cell death in the cortex and hippocampus regions of the brain. l-theanine also inhibited Aβ(1–42)-induced ERK and p38 mitogen-activated protein kinase along with the activity of nuclear factor kappa B (NF-kappa B), togethershowing that the positive effects of l-theanine on memory might be mediated by suppression of ERK/p38 and NF-kappa B, as well as through the reduction of macromolecular oxidative damage [81].

### 3.6. Alleviating Aβ-Induced Mitochondria Disfunction

l-theanine (a special amide found in tea leaf), EGCG, and rutin from green and black tea extracts showed protective effects against mitochondrial impairment, a very early event in AD pathogenesis. As a result, therapeutics targeting improved mitochondrial function could be beneficial. l-theanine significantly affected regulating mitochondrial fusion proteins in SH-SY5Y (APP(sw)) cells. Its possible molecular mechanism might be via its suppression of the abnormal expression of Mfn1 and Mfn2 caused by excessive intracellular Aβ [82].

Aβ induces mitochondrial dysfunction and synaptic impairments via production of ROS, which plays a role in the onset and progression ofAD. EGCG was identified as a mitochondrial restorative compound. EGCG treatment in an Aβ PP/PS-1 (presenilin 1) double mutant transgenic mice with AD restored mitochondrial respiratory rates, MMP, ROS production, and ATP levels by 50–85% in mitochondria isolated from the hippocampus, cortex, and striatum [83]. Aβ treatment increased Bax and intracytoplasmic cytochrome C, a protein associated with the mitochondria-dependent pathway. EGCG blocked the effect of Aβ-induced Bax increase, showing a protective effect against Aβ-induced neurotoxicity via inhibition of the expression of the protein associated with the mitochondria-dependent cell death pathway [84]. EGCG has the potential to protect neuronal mitochondrial function in AD.

Rutin is a component in green tea that can mitigate mitochondrial damage by alleviating oxidative stress and modulate the production of proinflammatory cytokines by decreasing TNF-α and IL-1β generation in microglia [85]. Black tea extract inhibited permeation of mitochondrial membranes induced by aggregate complexes of Aβ(42) and α-syn [86].

### 3.7. Inhibiting Hyperphosphorylation of TAU Protein

The accumulation of Aβ and TAU (a highly soluble microtubule-associated protein on the chromosome) aggregates is another pathological hallmark of AD. These polypeptides form fibrillar deposits and toxic oligomeric aggregation intermediates. Depleting these structures could therefore be a powerful therapeutic strategy for AD. GTP pretreatment reduced the hyperphosphorylated TAU protein in mice, showing neuroprotection against OA-induced neurotoxicity [36]. EGCG enhanced the clearance of phosphorylated TAU species in a highly specific manner byincreasing adaptor protein expression [87]. Both i.p. and orally-treated Tg mice were found to have modulated TAU profiles, with markedly suppressed sarkosyl-soluble phosphorylated TAU isoforms [37]. A test on a sporadic AD transgenic mouse model, known as senescence accelerated mouse prone 8 (SAMP8), showed that administration of EGCG could improve recognition and memory function by reducing Aβ and TAU hyperphosphorylation. Long-term oral consumption of EGCG at a relatively high dose (15 mg/kg) improved memory function in the SAMP8 mice in the Y-maze and Morris water maze. EGCG treatment also prevented the hyperphosphorylation of TAU and reversed the decreased synaptic protein marker synaptophysin and postsynaptic density protein 95 in the FC and hippocampus (Hip) of SAMP8 mice, accompanied by a significant decrease in the levels of Aβ(1–42) and BACE-1 activity. Long-term oral administration of EGCG may reduce the impairments in spatial learning and memory and decrease the reduction in synaptic proteins observed in an AD mouse mode [88].

TAU fragments (His-K18 δK280) formed toxic oligomeric aggregation intermediates individually or by interaction with Aβ. EGCG inhibited the aggregation of TAU (His-K18 δK280) into toxic oligomers at ten- to hundred-fold sub-stoichiometric concentrations, resulting in rescuing toxicity in neuronal model cells [89].

## 4. Conclusion and Future Challenges

**Figure 1:**
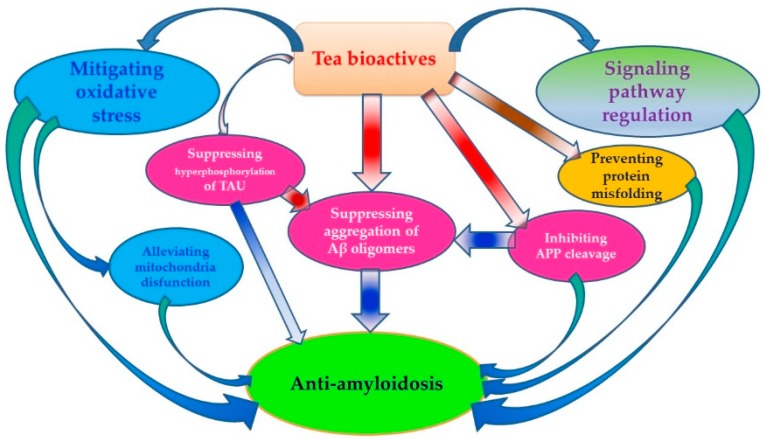
Anti-amyloidosis effects of tea.

In the beginning half of this review, we outlined the epidemiological evidence showing how tea consumption in many different regions of the world has been associated with either a decreased risk of neurodegenerative disease AD or an improvement in cognitive function in older populations. In the second half of this review, we discussed the numerous mechanisms by which the bioactive components in tea (EGCG, ECG, EGC, EC, l-theanine, and rutin) have anti-amyloid effects, thereby resulting in protection against AD. The anti-amyloid mechanisms of these bioactive compounds include: (1) inhibiting APP cleavage by regulating the activity of related enzymes, (2) preventing protein misfolding and membrane damage induced by Aβ, (3) mitigating Aβ-induced oxidative stress, (4) suppressing the aggregation of Aβ oligomers, (5) regulating signaling pathways involving Aβ generation, (6) reducing Aβ-induced mitochondria disfunction, and (7) inhibiting hyperphosphorylation of TAU protein (Figure 1).

Additional research will be required before we can affirmatively support a link between tea consumption and the prevention of cure for AD. Specifically, more clinical studies are needed to help clarify inconsistent epidemiological results [17,31,34]. Factors causing inconsistencies include poor stability of tea bioactive components [90], dosage differences between in vitro and in vivo tests [91], low bioavailability [92], and conversion of bioactivities in the gastrointestinal track [93,94]. In-depth studies on these factors will be of significance for bridging the gap between in vitro studies and clinical applications.

## Figures and Tables

**Table 1:** Epidemiological evidence for the association between tea intake and the risk of Alzheimer’s disease (AD) and related cognitive decline.

Type of Study	Country	Number of Subjects	Main Results	Reference
Six-year follow up longitudinal study	U.K.	Nine community-dwelling men and women.	The action-based memory of people with dementia of AD can be judged by looking at the process of preparing a cup of tea.	Rusted et al., 2002 [12]
Cross-sectional study	Japan	1003 Japanese subjects aged 70 or older.	Consumption of ≥2 cups/day green tea was associated with a lower prevalence of CoI (OR: 0.46 (95% CI: 0.30, 0.72; *p* = 0.0006), compared to reference (≤3 cups/week)	Kuriyama et al., 2006 [13]
Prospective cohort study	Japan	13,988 Japanese subjects aged 65 or older.	Green tea consumption was significantly associated with a lower risk of incident functional disability, even after adjustment for possible confounding factors.	Tomata et al., 2012 [14]
Population-based prospective study	Japan	490 Japanese residents over 60 years old.	The multiple adjusted OR for the incidence of dementia was 0.26 (95% CI: 0.06, 1.06) among individuals who consumed green tea every day compared with those who did not consume green tea at all. No association was found between coffee or black tea consumption and the incidence of dementia or MCI.	Noguchi-Shinohara et al., 2014 [15]
Cross-sectional study	Japan	1143 subjects.	Low green tea consumption (*p* = 0.032) were independently associated with a higher prevalence of CoI. The OR for drinking tea every day was 0.65 (95% CI: 0.47, 0.89)	Kitamura et al., 2016 [16]
A double-blind, randomized controlled study	Japan	33 nursing home residents, consumed 2 g/day of green tea powder for 12 months.	Cognitive disfunction was not significantly different compared with that of the placebo group (OR: −0.61 (95% CI: −2.97, 1.74), *p* = 0.59).	Ide et al., 2016 [17]
Cross-sectional study	Singapore	2501 adults aged 55 or older.	Cognitive decline ORs were 0.74 (95% CI: 0.54, 1.00) for low level, 0.78 (95% CI: 0.55, 1.11) for medium level, and 0.57 (95% CI: 0.32, 1.03) for high level tea intake.	Ng et al., 2008 [18]
Cross-sectional study	Singapore	716 adults aged 55 or older.	Total tea consumption was independently associated with better performance on global cognition, memory, executive function, and information processing speed.	Feng et al., 2010 [19]
Longitudinal aging study	Singapore	1615 adults aged 55 to 93.	The ORs were 0.79 (95% CI: 0.42, 1.48) for low tea consumption participants, 0.47 (95% CI: 0.25, 0.88) for medium tea consumption participants and 0.27 (95% CI: 0.11, 0.63) for high tea consumption participants (*p* = 0.01).	Feng et al., 2012 [20]
Cohort study	Singapore	614 elderly aged 60 or older who were free of dementia and cognitive impairment.	Long-term tea consumption was associated with reduced depressive and anxiety symptoms among community-living elderly.	Chan et al., 2017 [21]
Cohort study	China	681 unrelated Chinese nonagenarians/centenarians (67.25% women).	Habits of tea drinking had a significantly positive impact on CoI in men, but no association of CoI with tea drinking in women.	Huang et al., 2009 [22]
Population-based, nest case-control study	China	5691 elderly residents aged 65 or older (1489 cognitive decline and 4822 normal cognitive function).	An inverse association between tea drinking and cognitive decline was found (OR: 0.82; 95% CI: 0.69, 1.00, *p* = 0.0468).	Chen et al., 2012 [23]
Population-based survey	China	4579 elders aged 60 or older from the town of Weitang in Suzhou, China.	An inverse association was found between tea consumption (of any type) and prevalence of CoI (OR: 0.74, 95%CI: 0.57–0.98, *p* = 0.032).	Gu et al., 2017 [24]
Population-based study	China	1368 rural community-dwelling individuals aged 60 or older (59.3% female).	Daily tea consumption was associated with a lower likelihood of depressive symptoms in older Chinese people living in a rural community. The association appears to be independent of cerebrovascular disease and atherosclerosis.	Feng et al., 2013 [25]
Cross-sectional Study	China	9375 adults aged 60 or older.	An inverse correlation was found between tea consumption and prevalence of CoI.	Shen et al., 2015 [26]
Cross-sectional study	China	2015 adults aged 65 or older (42.2% men).	Tea consumption was associated with low prevalence of AD.	Yang et al., 2016 [27]
Longitudinal Healthy Longevity Survey	China	32,606 subjects aged 65 or older (13,429 men and 19,177 women).	High frequency of tea consumption was significantly associated with reduced OR of CoI.	Qiu et al., 2012 [28]
Longitudinal Healthy Longevity Survey	China	7139 participants aged 80 to 115 years.	Regular tea drinking was associated with better cognitive function in oldest-old Chinese, with regression coefficient 0.72 (*p* < 0.0001) for daily drinking and 0.41(*p* = 0.01) for occasional drinking.	Feng et al., 2012 [29]
Prevalence survey	China	1000 residents aged ≥60 years old.	Drinking tea reduced the incidence of MCI (*p* < 0.05)	Yang et al., 2017 [30]
Cross-sectional study	China	870 elders aged ≥90 years old.	Among the Chinese nonagenarians and centenarians, no significant correlation between tea consumption and the prevalence of MCI.	Wang et al., 2010 [31]
Cross-sectional study	Norway	2031 adults aged 70–74 years (55% women).	The associations between intake of tea and cognition were approximately linearly dose-dependent.	Nurk et al., 2009 [32]
Population-based study	23 developed countries	Adults from 23 developed countries and given different genetic backgrounds.	A significant inverse correlation was found between dietary consumption of flavonoids and rate of AD or related dementias.	Beking et al., 2010 [33]
Meta-analyses	Asia, Europe, Australia, and North America.	52,503 participants distributed in Asia, Europe, Australia, and America.	Daily tea drinking was associated with decreased risk of CoI, MCI andcognitive decline in the elderly. However, the association between tea intake and AD remained elusive.	Ma et al., 2016 [34]
